# Complex medium supplements optimized for the reduction of animal-derived components in vaccine production media

**DOI:** 10.1186/1753-6561-5-S8-P24

**Published:** 2011-11-22

**Authors:** James F Babcock, Karen A Benedict, Amanda L Perlman

**Affiliations:** 1 Sheffield Center for Cell Culture Technology, Sheffield Bio-Science, A Kerry Group Business, Ithaca, NY USA

## Introduction

A series of cell-line specific complex media supplements have been developed for enhancing performance of various vaccine production systems. Created using in-house media optimization methods, these supplements are manufactured using innovative process technology which allows for the formulation of complex and/or chemically defined animal-component free media additives into a single homogenized functional supplement. These supplements have been optimized for individual cell lines, and have proven to be suitable for use in a range of basal media. Data are presented which demonstrate the effectiveness of these optimized supplements for application as performance enhancers, and as a vehicle for serum-reduction in CEF, MDCK and BHK culture media. While these particular supplements do contain some undefined components, preliminary research indicates that this same technology may be applied to chemically-defined, multi-component supplements.

## Materials and methods

CEF and MDCK cells were maintained in T-75 flasks with a working volume of 15 ml. CEF cells were grown in DMEM + 5% Tryptose Phosphate Broth (TPB) + 5% FBS. MDCK cells were grown in DMEM + 10% FBS.

To generate growth curves for both CEF and MDCK, triplicate T-25 flasks were seeded at 2.0 x10^5^ cells/ml with a working volume of 7.5 ml. Cells were incubated at 37°C in 5% CO_2_ . One set of triplicates was set up for each day cells were to be counted. On days 3-6, one set of triplicates was trypsinized and re-suspended in fresh medium for counting.

BHK cultures were grown in 125 ml shake-flasks containing a final medium volume of 35 ml. The basal medium consisted of DMEM plus 10% FBS. Triplicate cultures were seeded at 2.0 x10^5^ cells/ml, and incubated at 37°C in 5% CO_2_ at 130 rpm for 12 days. Hydrolysate supplementation was achieved via the use of filter-sterilized 100 g/l stock solutions prepared in the basal medium.

On days 3-7, 1.0 ml of the culture supernatants were removed for assessing cell counts and viability. All cells were counted using a Nova BioProfile Flex automated cell counter.

## Results

**Figure 1:**
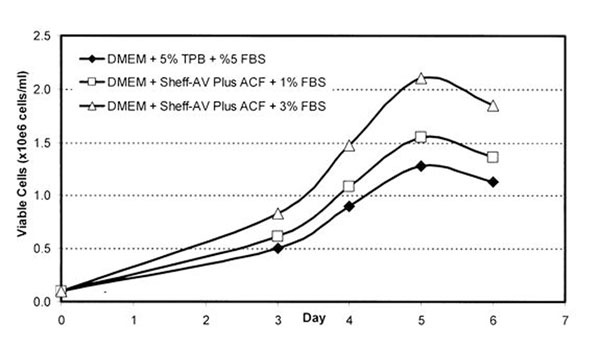
Growth performance of an animal component free medium supplement in Chicken Embryo Fibroblast (CEF) adherent cell culture

In a medium for Chicken Embryo Fibroblast (CEF) cells, incorporation of non-animal components into a complex supplement allows for reduction in the final concentration of serum in the basal medium formulation. This results in a significant reduction in animal-derived products as compared to the traditional CEF medium formulation containing 5%TBP + 5% FBS. The non-animal supplement out-performs the conventional medium with respect to cell growth when the serum level is reduced to 1% (Figure 1).

As with the CEF cells, various hydrolysates were screened to determine the most compatible with MDCK cells. Sheff-VAX is a hydrolysate-based animal component free supplement which can be employed to reduce FBS concentration from 10% to 3% or less. When Sheff-VAX is used in conjunction with 3% FBS, higher cell densities are achieved than with the control medium containing 10% FBS.

In BHK-21 culture, supplementation with Sheff-VAX or Sheff-VAX Plus ACF allows for significantly improved cell counts while simultaneously reducing the serum requirement. The control media for both cell lines contain 10% FBS, and both cell lines reached similar maximum cell densities. However, the magnitude of improvement achieved with the animal component free supplements was significantly greater with the BHK-21 cells as compared to the MDCK cells.

## Summary

The data presented illustrate how complex animal component free supplements may be employed to reduce or eliminate serum from many traditional vaccine production media, while providing equal or better performance with respect to cell growth. Partial or complete serum elimination was achieved in media used to cultivate CEF, MDCK and BHK-21 cell lines. These supplements have also proven to facilitate the transition from anchorage dependent to suspension culture.

